# RETRACTED: Gravina et al. The Small Molecule Ephrin Receptor Inhibitor, GLPG1790, Reduces Renewal Capabilities of Cancer Stem Cells, Showing Anti-Tumour Efficacy on Preclinical Glioblastoma Models. *Cancers* 2019, *11*, 359

**DOI:** 10.3390/cancers18010135

**Published:** 2025-12-31

**Authors:** Giovanni Luca Gravina, Andrea Mancini, Alessandro Colapietro, Simona Delle Monache, Roberta Sferra, Flora Vitale, Loredana Cristiano, Stefano Martellucci, Francesco Marampon, Vincenzo Mattei, Filip Beirinckx, Philippe Pujuguet, Laurent Saniere, Giocondo Lorenzon, Ellen van der Aar, Claudio Festuccia

**Affiliations:** 1 Division of Radiation Oncology, Department of Biotechnological and Applied Clinical Sciences, University of L’Aquila, Via Vetoio (Coppito II), 67100 L’Aquila, Italy; giovanniluca.gravina@univaq.it; 2 Laboratory of Radiobiology, Department of Biotechnological and Applied Clinical Sciences, University of L’Aquila, Via Vetoio (Coppito II), 67100 L’Aquila, Italy; mancio_1982@hotmail.com (A.M.); alecolapietro@gmail.com (A.C.); 3 Laboratory of cellular pathology, Department of Biotechnological and Applied Clinical Sciences, University of L’Aquila, Via Vetoio 1 (Coppito II), 67100 L’Aquila, Italy; simona.dellemonache@univaq.it (S.D.M.); s.martellucci@sabinauniversitas.it (S.M.); 4 Laboratory of Human Anatomy; Department of Biotechnological and Applied Clinical Sciences, University of L’Aquila, Via Vetoio (Coppito II), 67100 L’Aquila, Italy; Roberta.sferra@univaq.it; 5 Laboratory of Neurophysiology, Department of Biotechnological and Applied Clinical Sciences, University of L’Aquila, Via Vetoio (Coppito II), 67100 L’Aquila, Italy; floravitale86@Hotmail.it; 6 Department of Life, Health and Environmental Sciences, University of L’Aquila, Via Vetoio (Coppito II), 67100 L’Aquila, Italy; Loredana.cristiano@univaq.it; 7 Laboratory of Experimental Medicine and Environmental Pathology, University Hub “Sabina Universitas”, 02100 Rieti, Italy; vincenzo.mattei@uniroma1.it; 8 Department of Radiological, Oncological and Pathological Sciences, Sapienza University of Rome, 00161 Rome, Italy; f.marampon@gmail.com; 9 Galapagos NV, Industriepark Mechelen Noord, General De Wittelaan L11 A3, 2880 Mechelen, Belgium; filip.beirinckx@glpg.com (F.B.); Giocondo.lorenzon@glpg.com (G.L.); Ellen.vanderAar@glpg.com (E.v.d.A.); 10 Galapagos SASU, 102 avenue Gaston Roussel, 93230 Romainville, France; philippe.pujuguet@glpg.com (P.P.); laurent.saniere@glpg.com (L.S.)

The journal retracts the article, “The Small Molecule Ephrin Receptor Inhibitor, GLPG1790, Reduces Renewal Capabilities of Cancer Stem Cells, Showing Anti-Tumour Efficacy on Preclinical Glioblastoma Models” [1], cited above.

Following publication, concerns were brought to the attention of the Editorial Office regarding the integrity of an image presented in this publication [1].

Adhering to our standard procedure, an investigation was conducted by the Editorial Office and the Editorial Board that identified indications of partial overlap between the sub-images in Figure 1C (T24 and T28) published in [1] and overlap between Figure 5B published in [1] and Figure 4B published in [2], produced by a similar author group presenting different experimental conditions. While the authors provided replacement images during the investigation, the Editorial Board found the explanations regarding the duplicated data insufficient to warrant a correction. Consequently, the Editorial Board has lost confidence in the reliability of the findings and decided to retract this publication [1], as per MDPI’s retraction policy (https://www.mdpi.com/ethics#_bookmark30).

This retraction was approved by the Editor-in-Chief of the *Cancers* journal.

The authors did not provide a comment on this decision.

The corresponding author, Claudio Festuccia, takes full responsibility for the concerns raised and clarifies that the other co-authors were not directly involved in the duplication of the figures in question.
